# Dietary Fructus Schisandrae extracts and fenofibrate regulate the serum/hepatic lipid-profile in normal and hypercholesterolemic mice, with attention to hepatotoxicity

**DOI:** 10.1186/1476-511X-11-120

**Published:** 2012-09-19

**Authors:** Si-Yuan Pan, Qing Yu, Yi Zhang, Xiao-Yan Wang, Nan Sun, Zhi-Ling Yu, Kam-Ming Ko

**Affiliations:** 1 Department of Pharmacology, Beijing University of Chinese Medicine, Beijing, 100102, China; 2 School of Chinese Medicine, Hong Kong Baptist University, Hong Kong, China; 3 Division of Life Science, Hong Kong University of Science & Technology, Clear Water Bay, Hong Kong, China

**Keywords:** Fructus schisandrae chinensis, Fenofibrate, Fatty liver, Hypercholesterolemia, Hepatotoxicity, Hepatomegaly

## Abstract

**Background:**

Schisandra, a globally distributed plant, has been widely applied to health care products. Here, we investigated the effects of dietary intake of Fructus Schisandrae chinensis (FSC), both aqueous and ethanolic extracts (AqFSC, EtFSC), on serum/hepatic lipid contents in normal diet (ND)- and high-fat/cholesterol/bile salt diet (HFCBD)-fed mice.

**Methods:**

Male ICR mice were fed with ND or HFCBD, supplemented with 1 and 4% of AqFSC and EtFSC, respectively, or 0.1% fenofibrate, for 13 days. Lipids were determined according to the manufacture’s instructions.

**Results:**

EtFSC, but not AqFSC, significantly elevated hepatic triglyceride (TG) in mice fed with ND. Feeding mice with HFCBD increased serum total cholesterol (TC), high density lipoprotein (HDL) and low density lipoprotein (LDL) levels as well as alanine aminotransferase (ALT) activity. Supplementation with AqFSC, EtFSC or fenofibrate significantly reduced hepatic TC and TG levels. However, AqFSC and EtFSC supplementation increased serum HDL and LDL levels in mice fed with HFCBD. Fenofibrate increased serum HDL and reduced serum LDL contents in hypercholesterolemic mice. EtFSC reduced, but fenofibrate elevated, serum ALT activity in both normal and hypercholesterolemic mice. While fenofibrate reduced serum TC, TG, and HDL levels in mice fed with ND, it increased serum HDL and reduced serum LDL and TC levels in mice fed with HFCBD. Hepatomegaly was found in normal and hypercholesterolemic mice fed with diet supplemented with fenofibrate.

**Conclusions:**

Feeding mice with AqFSC and EtFSC ameliorated the HFCBD-induced hepatic steatosis. In addition, EtFSC may offer protection against hepatic injury in hypercholesterolemic mice.

## Background

Increasing incidence of coronary heart disease (CHD) and peripheral vascular disease as well as fatty liver is a result of hyperlipidemia caused by unhealthy lifestyle. The epidemic is also partly due to the failure in introducing effective therapeutic intervention for hyperlipidemia and hepatoteatosis. Hepatosteatosis, which is also called nonalcoholic fatty liver disease (NAFLD), is characterized by lipid deposition within hepatocytes in patients having no history of excessive alcohol consumption. Being coined as a refractory progressive disease, NAFLD can develop into liver cirrhosis or hepatocellular carcinoma, as well as nonalcoholic steatohepatitis (NASH) [1-3]. Therefore, the search for therapeutic interventions aimed at lowering lipid contents in blood and liver has been an area of intensive research. Drugs that are currently available for lowering blood lipids reduced the coronary artery disease mortality of 23% and cardiovascular disease mortality of 19% in patients with dyslipidemia [4]. However, there are no effective drugs or therapeutic strategies for the prevention of NAFLD in patients suffering from hyperlipidemia. While chemical drugs are less appealing for their potential adverse side effects, the use of naturally-occurring ingredients, such as soy isoflavones, soy foods, prebiotic fibres, pentoxifylline and silibinin and plant medicine for prevention and/or treatment of NAFLD and dyslipidemia has become increasingly popular [5-9].

Schisandra genus of about 25 species throughout the world, at present, it are mainly distributed in northern China. Fructus Schisandrae chinensis (FSC), which is the fruit of *Schisandra chinensis*, is a traditional Chinese herb used for thousands of years in China. Modern scientific research has shown that FSC extract or its active ingredient (schisandrin B) and other related compounds (bifendate, and bicyclol) possess a wide spectrum of biological activities, particularly in protecting against chemically- and virally-induced hepatitis [10-13]. In addition, previous studies in our laboratory have shown that both FSC extract and the active compounds (schisandrin B, bifendate and bicyclol) reduced hepatic triglyceride (TG) and total cholesterol (TC) levels in mice with hypercholesterolemia produced by high-fat diet containing cholesterol/bile salt [14-17]. In the present study, we endeavored to investigate the effects of dietary supplementation with aqueous or ethanolic extract of FSC on serum and hepatic lipid contents, with attention to hepatotoxicity, in normal and hypercholesterolemic mice. Fenofibrate was also used as a positive control for comparison. The objective of this study is to establish a pharmacological basis for the potential application of FSC in the treatment of NAFLD and NASH.

## Results

### Serum lipid profiles

Supplementation with AqFSC and EtFSC at a dose of 1 or 4% did not affect serum levels of TC, TG, HDL, and LDL in mice fed with ND. However, both AqFSC and EtFSC significantly increased serum HDL and LDL levels (by 6–13% and 31–47%, respectively) in hypercholesterolemic mice. But they did not affect the serum TC and TG levels. HFCBD markedly increased serum TC, HDL and LDL levels (by 119, 19, and 494%, respectively) in mice. However, the serum TG level was decreased by 74%. On the other hand, fenofibrate treatment reduced serum TC (31%), TG (29%), and HDL (32%) levels in normal mice. While the serum HDL level was elevated by 29%, TC and LDL levels were reduced by 39 and 57%, respectively, in mice fed HFCBD/fenofibrate-supplemented diet, when compared with those fed with HFCBD only (Table 1).

**Table 1:** Effects of dietary supplementation with AqFSC, EtFSC, or fenofibrate on serum lipid profiles in normal and hypercholesterolemic mice

**Groups**	**TC (mmo1/L)**	**TG (mmo1/L)**	**HDL (mmo1/L)**	**LDL (mmo1/L)**
***Normal diet for 13 days***				
Normal diet	5.13 ± 0.18	1.84 ± 0.11	4.14 ± 0.14	0.33 ± 0.02
1% AqFSC/normal diet	4.90 ± 0.13	1.84 ± 0.08	4.13 ± 0.12	0.38 ± 0.04
4% AqFSC/normal diet	4.86 ± 0.16	2.03 ± 0.12	4.13 ± 0.16	0.38 ± 0.03
1% EtFSC/normal diet	4.89 ± 0.21	1.73 ± 0.07	4.01 ± 0.16	0.38 ± 0.02
4% EtFSC/normal diet	4.89 ± 0.17	1.77 ± 0.10	4.00 ± 0.12	0.39 ± 0.02
0.1% Fenofibrate/normal diet	3.54 ± 0.34*	1.30 ± 0.12*	2.80 ± 0.18*	0.39 ± 0.03
**HFCB diet for 13 days**				
Normal diet	3.97 ± 0.24	1.19 ± 0.15	3.86 ± 0.12	0.33 ± 0.03
HFCB diet	8.71 ± 0.82*	0.31 ± 0.04*	4.59 ± 0.11*	1.96 ± 0.16*
1% AqFsSC/HFCB diet	7.01 ± 0.31	0.66 ± 0.05	5.17 ± 0.25^†^	2.87 ± 0.24^†^
4% AqFSC/HFCB diet	6.62 ± 0.24	0.27 ± 0.03	5.16 ± 0.18^†^	2.56 ± 0.14^†^
1% EtFSC/HFCB diet	8.63 ± 0.68	0.26 ± 0.03	4.87 ± 0.14^†^	2.68 ± 0.10^†^
4% EtFSC/HFCB diet	9.35 ± 0.37	0.27 ± 0.03	5.16 ± 0.12^†^	2.89 ± 0.27^†^
0.1% Fenofibrate/HFCB diet	5.30 ± 0.21^†^	0.67 ± 0.04	5.91 ± 0.32^†^	0.85 ± 0.15^†^

Mice were fed with either normal or high-fat/cholesterol/bill salt (HFCB, 10/1/0.3%, w/w) diet without and with supplementation with the aqueous/ethanolic extract of Fructus Schisandrae chinensis (AqFSC/EtFSC) or fenofibrate at the indicated concentration for 13 days. The concentrations of AqFSC and EtFSC were based on crude herbal material. Serum total cholesterol (TC), triglyceride (TG), high density lipoprotein (HDL), and low density lipoprotein (LDL) levels were then measured. Values given are the men ± S.E.M., with n = 10. *Significantly different from the normal diet group (*p* < 0.05–0.001). ^†^significantly different from the hypercholesterolemic mice (*p* < 0.05–0.001).

### Hepatic lipid contents and hepatic index

**Figure 1:**
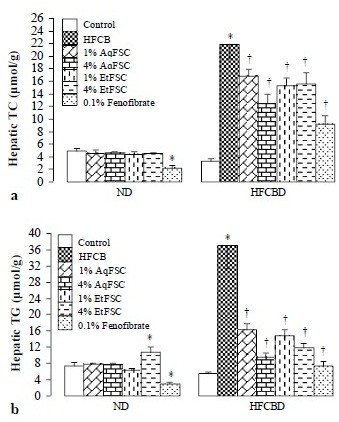
**Effects of dietary supplementation with AqFSC, EtFSC, or fenofibrate on hepatic lipid contents in normal and hypercholesterolemic mice.** Mice were fed with a normal or high-fat diet without and with supplementation with the drugs for 13 days. Experimental details were described in Table 1. Hepatic total cholesterol (TC) and triglyceride (TG) levels were then measured. Values given are the mean ± S.E.M., with n = 10. ^*^Significantly different from the normal diet group (*p* < 0.05–0.001); ^†^significantly different from the hypercholesterolemic mice (*p* < 0.05–0.001).

**Figure 2:**
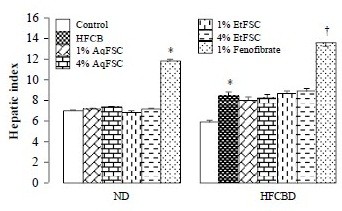
**Effects of dietary supplementation with AqFSC, EtFSC, or fenofibrate on hepatic weight in normal and hypercholesterolemic mice.** Mice were fed with a normal or high-fat diet without and with supplementation with the drugs for 13 days. Experimental details were described in Table 1. Hepatic index was estimated from the ratio of total liver weight to body weight. Values given are the mean ± S.E.M., with n = 10. ^*^Significantly different from the normal diet group (*p* < 0.001); ^†^significantly different from the hypercholesterolemic mice (*p* < 0.001).

Feeding mice with 4% EtFSC with ND for 13 days significantly increased hepatic TG content by 46%. While 0.1% fenofibrate with normal diet reduced hepatic TC and TG contents (by 56 and 60%, respectively), it increased the hepatic index by 70%. Feeding mice with HFCBD markedly increased hepatic TC and TG contents as well as liver weight (by 560, 566, and 41%, respectively), when compared with those of mice fed with ND. Supplementation with AqFSC or EtFSC at concentration of 1 or 4% decreased the hepatic TC and TG contents by 23–43 and 56–74%, respectively, in HFCBD-fed mice. Fenofibrate treatment reduced hepatic TC and TG contents (by 58 and 80%, respectively) in HFCBD-fed mice, but it increased the hepatic index by 62% (Figures 1 and 2).

### Hepatotoxicity

**Figure 3:**
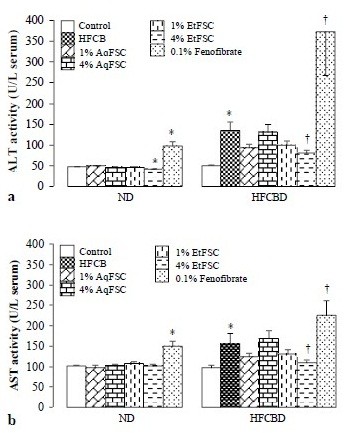
**Effects of dietary supplementation with AqFSC, EtFSC, or fenofibrate on liver function in normal and hypercholesterolemic mice.** Mice were fed with a normal or high-fat diet without and with supplementation with the drugs for 13 days. Experimental details were described in Table 1. Serum alanine aminotransferase (ALT) and aspartate aminotransferase (AST) activity were then measured. Values given the mean ± S.E.M., with n = 10. ^*^Significantly different from the normal diet group (*p* < 0.05–0.001); ^†^significantly different from the hypercholesterolemic mice (*p* < 0.05–0.001).

Feeding mice with diet supplemented with 4% EtFSC lowered serum ALT, but not AST, activity (up to 13%) (*p* < 0.05) in normal mice. HFCBD caused increase in serum ALT (by 169%) and AST (by 61%) activities in mice. Supplementation with EtFSC, but not AqFSC, decreased serum ALT and AST activity (up to 40%) in HFCBD-fed mice. Dietary supplementation with fenofibrate increased serum ALT and AST activities in mice fed with ND and HFCBD diet (Figure 3).

### Weigh gain and food/drug intake

No detectable differences in body weight gain between AqFSC/EtFSC supplemented and unsupplemented mice fed with ND and HFCBD were observed. However, weight loss was observed in fenofibrate supplemented mice with ND (by 17.2%) and HFCBD (by 12.5%). Daily intake of AqFSC/EtFSC was estimated to be 1.35–1.99 g/kg (based on crude herb equivalent) at 1% supplementation and 5.43–6.68 g/kg at 4% supplementation (Table 2). The daily intake of fenofibrate was estimated to be 0.17 g/kg.

**Table 2:** Effects of dietary supplementation of AqFSC, EtFSC, or fenofibrate on body weight gain and food/drug intake in the normal and hypercholesterolemic mice

**Groups**	**Body weight gain (g)**	**Body-liver weight (g)**	**Food intake (g/mouse/day)**	**Drug intake (g/kg/day)**
***Normal diet for 13 days***				
Normal diet	8.55 ± 0.11	24.64 ± 0.36	4.20	
1% AqFSC/normal diet	8.11 ± 0.19	24.63 ± 0.63	4.55	1.45
4% AqFSC/normal diet	8.05 ± 0.34	24.15 ± 0.58	4.32	5.56
1% EtFSC/normal diet	8.31 ± 0.15	24.53 ± 0.43	4.43	1.35
4% EtFSC/normal diet	8.45 ± 0.25	24.38 ± 0.43	4.21	5.43
0.1% Fenofibrate/normal diet	5.09 ± 0.08*	20.40 ± 0.28*	4.53	0.18
***HFCB diet for 13 days***				
Normal diet	8.00 ± 0.43	25.72 ± 0.63	4.31	-
HFCB diet	7.52 ± 0.25	24.96 ± 0.54	3.85	-
1% AqFSC/HFCB diet	8.23 ± 0.67	24.76 ± 0.59	4.56	1.59
4% AqFSC/HFCB diet	7.96 ± 0.42	25.49 ± 0.63	4.89	6.68
1% EtFSC/HFCB diet	8.80 ± 0.35	25.29 ± 0.76	5.50	1.99
4% EtFSC/HFCB diet	8.71 ± 0.52	25.25 ± 0.72	4.23	6.13
0.1% Fenofibrate/HFCB diet	7.33 ± 0.32	22.51 ± 0.72^†^	4.31	0.17

Experimental details were described in Table 1. The dosages (g/kg/day) based on crude herbal material were determined with respect to the amount of ingested diet (g/day/kg) and drug concentrations in the diet. Values given are the mean ± S.E.M., with n = 10. *Significantly different from the normal diet group (*p* < 0.01); ^†^significantly different from the hypercholesterolemic mice (*p* < 0.01).

### Acute toxicity

In the acute toxicity test, either AqFSC or EtFSC was administered intragastrically at increasing doses (24.5–71.6 g/kg) and the mortality rate was determined within 48 h post-dosing in mice. LD_50_ value of AqFSC and EtFSC was estimated to be 43.24 ± 7.79 g/kg and 40.35 ± 6.22, respectively, using the Bliss method.

## Discussion

Hyperlipidemia, which can be broadly categorized into hypercholesterolemia, hypertriglyceridemia and mixed hyperlipidemia, is causally related to the pathogenesis of CHD, cerebrovascular diseases, peripheral vascular disease and pancreatitis [18-21]. One major cause hyperlipidemia is unhealthy eating habit. In the present study, both serum and hepatic lipid levels were notably elevated in mice fed with HFCBD, a high-fat diet used in the experiment. Although HDL levels are regulated by cholesteryl ester transfer protein, hepatic TG lipase and lipoprotein lipase etc., the increased HDL and LDL levels may be an event secondary to hypercholesterolemia induced by feeding HFCBD in mice. In this connection, Guay et al. found that the cholesterol-elevating effect of a high-fat diet was associated with the formation of larger LDL particles than those of a low-fat diet [22]. It is well known that LDL is formed from very LDL (VLDL) which secretes from liver and allows the supply of triglycerides to tissues. However, it is not clear as to why feeding mice with HFCBD resulted in low serum TG levels [14-16,23]. Lipogenic activity appears to be up-regulated in obese condition and serum triglyceride is consumed for lipid synthesis, so serum TG decreased, but the obese did not found in the mice fed with a high-fat diet. In clinical situation, blood TG levels in patients with hypercholesterolemia are not lower than those of normal individuals. Therefore, the mouse model of hypercholesterolemia, as adopted in the present study, may be different from hypercholesterolemia in human patients in the pathogenesis of the disease state. The apparent liver injury induced by feeding HFCBD is likely related to the accumulation of fat in hepatic tissue [24,25]. In addition, hyperlipidemia is usually concerned hyperglycemia and hyperinsulinemia caused by insulin resistance in clinic [26], but in the present study, serum glucose levels were no significant differences between model group and normal mice (data not shown). This may be related to the short-time modeling (13 days).

Fenofibrate, a fibric acid derivative, is used to treat severe hypertriglyceridemia and mixed dyslipidemia (i.e., increases both TC and TG levels in blood) in patients who did not respond to non-pharmacological intervention, of which the condition is usually associated with an increased risk of atherosclerosis and/or fatty liver [27]. Fenofibrate treatment was found to reduce serum TG and LDL levels, but increase HDL level in hyperlipidemic patients [28]. In the present study, fenofibrate supplementation decreased serum TC, TG, and HDL levels in ND-fed mice. The reduction of HDL level by fenofibrate may result from the drug-induced reaction secondary to hypolipidemic action [29]. In addition, liver injury and hepatomegaly induced or aggravated by fenofibrate treatment were observed in ND and HFCBD-fed mice. Although fenofibrate treatment improved the hepatic microcirculatory patency and oxygen availability in a high-fat diet-induced fatty liver in mice as well as suppressed the growth of human hepatocellular carcinoma cells *in vitro*, it caused DNA damage in rat livers and promoted hepatocarcinogenesis through increasing oxidative stress in rodents [30-33]. Furthermore the long-term and high-dose administration of fenofibrate caused liver damage and dysfunction in rodents [34-36]. Fenofibrate is available in a formulation of 100-mg tablet for oral use at a daily dose of 300 mg in adult patients (i.e., 5 mg/kg/day for 60 kg). The dose (180 mg/kg in ND-fed mice and 170 mg/kg in HFCBD-fed mice) adopted in the present study is about 35–fold higher than the human dose.

Despite recent advances in the understanding of hyperlipidemia and its associated adverse clinical outcomes, hyperlipidemia remains the major cause of morbidity and mortality throughout the world. While chemical drugs are potent in lowering lipid levels in blood, they also produce some side effects in patients who require life-long medication. Recently, a shift from drug therapy to dietary supplementation with naturally-occurring ingredients has become a trend in the management of hyperlipidemia and fatty liver disease. Medicinal plants have been used for over millennia and are highly esteemed all over the world as a rich source of therapeutic agents, food supplements and/or additives for the prevention and treatment of diseases. FSC, which is a commonly used Chinese herb, was tested in the mouse model of hypercholesterolemia. Although both AqFSC and EtFSC notably lowered hepatic TC and TG contents and elevated serum HDL and LDL levels in mice fed with HFCBD, no significant differences in serum lipid levels were observed in mice fed with normal diet supplemented with AqFSC and EtFSC. This suggested that the effect of FSC on lipids may be dependent on basal lipid levels in the body. Under normal conditions, FSC did not affect the serum lipid levels, but it exaggerated the elevation of serum HDL and LDL by HFCBD. It is well known that HDL and LDL are “good cholesterol” and “bad cholesterol” with respect to their role in the development of CHD [37-39]. Whether or not dietary FSC-induced increase in both HDL and LDL levels is “good” or “bad” remains to be investigated.

Both AqFSC and EtFSC alleviated the accumulation of lipids in livers of HFCB diet-fed mice, but only EtFSC suppressed the serum ALT activity, a biochemical index of liver damage. The inability of AqFSC to protect against liver damage in hypercholesterolemic mice may be related to the possibility that the anti-fatty liver ingredients, which are present in both AqFSC and EtFSC, are distinct from those for liver protection, with the latter being present in EtFSC only. As EtFSC also inhibited serum ALT activity in ND-fed mice, the direct inhibition of EtFSC on ALT activity cannot be excluded. The hepatoprotective effect of EtFSC can be confirmed by the measurement of alternative housekeeping enzymes like sorbitol dehydrogenase in the blood and/or histological analysis of liver tissue. Lignans are currently considered as hepatoprotective ingredients in FSC by virtue of their *in vivo* antioxidant potential [40,41]. The fat-soluble lignans likely reside in EtFSC rather than AqFSC [42,43]. Therefore lignans in FSC unlikely contribute to the amelioration of fatty liver induced by HFCBD. There are eight integrants in the water-soluble fraction of FSC through spectral analysis, they include protocatechuic acid, quinic acid, 2-methyl citrate, 5-hydroxymethyl-2-furancarboxaldehyde, zingerone glucoside, thymoquinol 2-glu-coside, thymoquinol 5-glucoside, daucosterol [44]. EtFSC mainly contains lignans including schizandrin A, B, and C; schizandrol A and B; gomisin B, C, D, E, G, H, J, and N; tigloylgomisin H; and angeloylgomisin H etc. [45].

Fenofibrate has been prescribed for patients with dyslipidemia in the US since 1998. The lipid-lowering action of fenofibrate in the blood (or likely in liver tissue too) has been shown to be mediated by the activation of PPAR-alpha and lipoprotein lipase, as well as suppression of apoliprotein C-III, fatty acid synthase, acetyl CoA carboxylase, and cholesterol absorption, etc. [46-49]. Recently, it is revealed that fenofibrate induces the activity of enzymes in the tricarboxylic acid cycle [50]. It is unclear whether this may contribute to weight loss induced by fenofibrate. Although active ingredient of FSC (schisandrin B) and its related analogs (bifendate, and bicyclol) have been shown to affect lipid metabolism [51-54], the mechanism underlying the lipid-lowering effect of AqFSC and EtFSC remains to be elucidated.

## Conclusion

Dietary supplementation with AqFSC or EtFSC and fenofibrate ameliorated the fatty liver condition in HFCBD-fed mice. However, both AqFSC and EtFSC increased serum HDL and LDL levels in HFCBD-fed mice. EtFSC, but not AqFSC, supplementation reduced serum ALT activity in both ND- and HFCBD-fed mice. Although fenofibrate produced a potent hypolipidemic action, it caused hepatotoxicity and hepatomegaly. Naturally-occurring ingredients from medicinal plants such FSC may be a safe alternative for the management of fatty liver.

## Materials and methods

### Plant material and extraction procedure

FSC, the fruit of *Schisandra chinensis* (Turcz.) Baill., was purchased from the Anguo Chinese herbs market and authenticated by Dr. H. Dong. For the preparation of the aqueous extract of FSC (AqFSC), 470 g of powdered FSC was boiled in ten volumes of distilled water for 1 h. The procedure was repeated twice with 8 volumes of water. The pooled aqueous extract was filtered through gauze cloth and the filtrate was evaporated by heating to obtain 200 ml of pre-AqFSC. The pre-AqFSC (200 ml) was then precipitated twice with ten and five volumes of 95% ethanol for 12 h at 5°C. Eighty-nine g of AqFSC (i.e., 5.28 g of herb for every 1 g of AqFSC) was finally obtained and stored at 4°C until use. For the preparation of the ethanolic extract of FSC (EtFSC), FSC was crushed into small pieces and extracted twice with five volumes of 80% (v/v, in H_2_O) ethanol under reflux. The pooled extract was filtered by filter paper and concentrated under reduced pressure by rota-evaporation to obtain EtFSC, with a yield of 50% (w/w) (i.e., 2 g of herb for every 1 g of extract), and stored at 4°C until use.

### Chemicals and reagents

Cholesterol (certificate no. 041103) and bile salt (certificate no. 000710) were obtained from Beijing Chemical Reagent Co. (Beijing, China). Fenofibrate (certificate no. 0405030) was bought from Beijing Yongkang Medical Ltd. (Beijing, China). Assay kits for hepatic TC and TG were bought from Zhongsheng Beikong Bio-technology and Science Inc. (Beijing, China). Serum TC, TG, high-density lipoprotein cholesterol (HDL), and low-density lipoprotein cholesterol (LDL) levels as well as alanine aminotransferase (ALT), and aspartate aminotransferase (AST) activities were measured using commercial kits from Beijing Leadman Biochemistry CO., Ltd. (Beijing, China).

### Animal treatment

Male ICR mice (Grade II, certificate No. SCXK(jing) 2006–0009), weighing 18–20 g, were supplied by Vital River Lab Animal Co. Ltd. (Beijing, China). All animals were maintained on a 12 h (light on 700–1900 h) light-dark cycle at 20–21, with a relative humidity of 50–55%. They were allowed free access to water and food. Animals were divided into 6 groups of 10 animals in each: (1) mice fed normal diet (ND); (2) and (3) mice fed diet supplemented with 1% (w/w) and 4% AqFSC, respectively; (4) and (5) mice fed diet supplemented with 1% and 4% EtFSC, respectively; and (6) mice fed diet supplemented with 0.1% fenofibrate. In addition, the effect of FSC extracts on mice with hypercholesterolemia induced by high-fat/cholesterol/bile salt diet (HFCBD, 10/1/0.3%, w/w) was also investigated. Seventy mice were randomized into seven groups of 10 animals in each: (1) mice fed ND; (2) mice fed HFCBD; (3)-(6) mice fed HFCBD supplemented with AqFSC or EtFSC diet at 1% and 4% concentrations; (7) mice fed HFCBD supplemented with fenofibrate at 0.1%. Mice were fed ND or HFCBD supplemented with AqFSC/EtFSC or fenofibate for 13 days. Then blood and liver tissue samples were obtained from ether-anesthetized animals which had been fasted for 6 h (from 600 to 1200). Hepatic index was estimated from the ratio of total liver weight to body weight. All experimental procedures were approved by the University Committee on Research Practice in Beijing University of Chinese Medicine.

### Biochemical analysis

Serum and liver samples were obtained 24 h after the last day of the experiment. Serum samples were prepared by centrifuging the whole blood obtained from the orbital vein for 8 min at 2000 × *g* and stored at −70°C until use for biochemical analyses. The liver tissue sample was homogenized in 9 volumes of 0.9% (w/v) NaCl solution by two 10-s bursts of a tissue disintegrator at 13,500 rpm, and the homogenate was then centrifuged at 2000 × *g* for 15 min to obtain the supernatants. Ten and 40 μl of the hepatic supernatant were used to determine the TG and TC levels, respectively, according to the manufacture’s instructions. Serum lipid profiles such as TC, TG, HDL, and LDL, as well as ALT and AST activities were determined using LM280 automatic clinical chemistry analyzer (Leadman Group Co., Ltd.)

### Statistical analysis

All values are expressed as means ± S.E.M. Data were analyzed by 1-way ANOVA using SPSS (version 12) statistical analysis program, and then differences among means were analyzed using Duncan’s test. *p* < 0.05 was considered significant.

## Abbreviations

NAFLD: Nonalcoholic fatty liver disease; NASH: Nonalcoholic steatohepatitis; FSC: Fructus Schisandrae chinensis; AqFSC: Aqueous extract of FSC; EtFSC: Ethanolic extract of FSC; TG: Triglyceride; TC: Total cholesterol; HDL: High-density lipoprotein cholesterol; LDL: Low-density lipoprotein cholesterol; ALT: Alanine aminotransferase; AST: Aspartate aminotransferase; CHD: Coronary heart disease.

## Competing interests

The authors declare that they have no competing interests.

## Authors’ contributions

Design of the study: SYP, ZLY; conduct of the study: QY, YZ; data collection: XYW, NS; data analysis: SYP; data interpretation: SYP, KMK; manuscript writing: SYP, KMK. All authors read and approved the final manuscript.

## Authors’ information

Si-Yuan Pan is a professor; Qing Yu, Yi Zhang, Xiao-Yan Wang and Nan Sun are Master Degree Graduate; Zhi-Ling Yu, PhD, is an associate professor; Kam-Ming Ko, PhD, is a professor.
